# Low‐Temperature, Sustainable Manufacturing of Printed OTFT Backplanes for Electrophoretic and OLED Displays

**DOI:** 10.1002/advs.202514091

**Published:** 2025-09-30

**Authors:** Yasunori Takeda, Miho Abiko, Kaori Watanabe, Ryoko Horie, Yasutaka Nakamura, Junghwi Lee, Shohei Yumino, Shinya Oku, Tomohito Sekine, Makoto Mizukami

**Affiliations:** ^1^ Innovation Center for Organic Electronics Yamagata University 1‐808‐48, Arcadia Yonezawa Yamagata 992‐0119 Japan; ^2^ Advanced Materials Research Laboratory Tosoh Corporation 2743‐1, Hayakawa Ayase Kanagawa 252‐1123 Japan; ^3^ Graduate School of Organic Materials Science Yamagata University 4‐3‐16, Jonan Yonezawa Yamagata 992‐8510 Japan; ^4^ Research Center for Organic Electronics (ROEL) Yamagata University 4‐3‐16, Jonan Yonezawa Yamagata 992‐8510 Japan

**Keywords:** active‐matrix backplane, electrophoretic display, low‐temperature process, organic thin‐film transistors, printed electronics

## Abstract

Printed electronics have garnered significant attention for developing lightweight, cost‐effective, and environmentally friendly systems, driving significant advancements in the realm of sustainable electronics. This study introduces a printed active‐matrix organic thin‐film transistor (OTFT) backplane, fabricated under ambient conditions using low‐temperature printing and solution‐based processes. All electrode layers—including gate, source/drain, and pixel electrodes—are patterned through reverse‐offset printing (ROP), achieving high‐resolution multilayer integration with minimum line widths of 10 µm. The resulting OTFTs demonstrate robust performance in ambient air, achieving a carrier mobility of 1.12 cm^2^ Vs^−1^ and on/off ratios exceeding 10⁸. The printed backplane is integrated with an electrophoretic display panel, enabling stable black‐and‐white image rendering through pixel‐level addressing. The same printing technology is employed to fabricate and drive an organic light‐emitting diode (OLED) display, utilizing white OLEDs with laminated color filters. X‐ray photoelectron spectroscopy validates that residual polydimethylsiloxane (PDMS) contamination from the ROP process can be eliminated through IPA rinsing, ensuring clean electrode surfaces. The fabrication process is conducted at temperatures exceeding 160 °C without vacuum equipment, significantly reducing energy consumption and material waste. This environmentally sustainable approach can be employed to develop future flexible and disposable electronics. The versatility of printed backplanes demonstrated here highlights their potential to drive next‐generation, environmentally friendly display technologies.

## Graphical abstract

This study reports the fabrication of organic transistor backplanes using reverse‐offset printing and solution processes at ≤ 160 °C under ambient conditions. The backplanes successfully drive both electrophoretic and organic light‐emitting diode (OLED) displays with reliable performance. The approach highlights a versatile platform for flexible displays while promoting sustainable, low‐energy manufacturing in printed electronics.

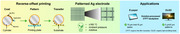

## 1 Introduction

Printed electronics have garnered significant attention as a promising approach for developing lightweight, cost‐effective, and environmentally friendly systems, driving significant advancements in the field of sustainable electronics.^[^ ^1^, ^2^, ^3^, ^4^, ^5^, ^6^ ^]^ Among the various applications, flexible displays—such as electronic paper (e‐paper) and organic light‐emitting diode (OLED) displays—are particularly noteworthy for their thin profiles, mechanical flexibility, and low power consumption, rendering them particularly well‐suited for integration into wearable and portable devices.^[^ ^7^, ^8^, ^9^, ^10^, ^11^ ^]^ However, fully realizing the potential of these technologies depends on the development of reliable and flexible backplane circuits that are compatible with printing‐based manufacturing processes.

Conventionally, display backplanes have been fabricated using photolithography and vacuum deposition techniques, which provide high resolution and excellent device performance.^[^ ^12^, ^13^ ^]^ However, these conventional methods are often complex, require costly equipment, and are not well‐suited for producing large‐area or flexible substrates.^[^ ^14^, ^15^, ^16^, ^17^ ^]^ Conversely, the advent of solution‐processable organic semiconductors, along with printable dielectric and conductive materials, has enabled the fabrication of printed backplanes on flexible substrates through additive manufacturing techniques, in combination with photopatterned dielectric and passivation layers.^[^ ^10^, ^11^ ^]^

Among the various device options available, organic thin‐film transistors (OTFTs) have emerged as particularly promising candidates for printed backplanes, owing to their low‐temperature processability, mechanical flexibility, and compatibility with diverse substrates.^[^ ^18^, ^19^, ^20^, ^21^, ^22^, ^23^ ^]^ However, fabricating high‐resolution, uniform OTFT arrays exclusively through printing remains a significant challenge, particularly for applications such as display driving circuits.^[^ ^24^, ^25^ ^]^

Printed and flexible backplanes have also been demonstrated using other classes of semiconductors, including solution‐processed metal‐oxide, printed carbon‐nanotube (CNT), and emerging 2D‐material TFTs. Each of these technologies presents unique trade‐offs in terms of mobility, operating voltage, processing temperature, and environmental stability.^[^ ^26^, ^27^, ^28^, ^29^, ^30^, ^31^, ^32^ ^]^ For example, metal‐oxide channels typically offer superior mobility and stability but often require plasma/UV‐ozone or high‐temperature treatments to optimize interfaces. CNT networks, on the other hand, enable low‐temperature processing and mechanical robustness, yet demand precise control over metallic content and contact resistance. Meanwhile, 2D semiconductors are highly attractive for ultimate scaling but face challenges related to wafer‐scale uniformity and integration with printing processes. In this context, our approach focuses on ambient‐pressure, low‐temperature manufacturing. We employ high‐resolution reverse‐offset printing (ROP) to define all metal electrodes, whereas UV‐photopatterned polymer layers facilitate multilayer integration and reliable via formation on flexible polyethylene naphthalate (PEN) substrates.

This study proposes the fabrication and functional validation of a printed OTFT backplane featuring photopatterned dielectric and passivation layers, specifically optimized for active‐matrix driving of electrophoretic displays. The gate and source/drain electrodes were patterned using ROP, a high‐resolution technique. Robust interlayer connectivity was achieved through the incorporation of finely structured via‐holes. The printed OTFTs utilize a bottom‐gate, bottom‐contact (BGBC) architecture and demonstrate an average mobility of ≈0.713 cm^2^ V·s^−1^. The fabricated backplane was successfully integrated with an electrophoretic display, enabling reliable pixel‐level switching. Notably, the same backplane architecture was also employed to drive an OLED display, demonstrating the versatility of the proposed approach.

## 2 Results and Discussion

### 2.1 Printing Process Development

**Figure 1:**
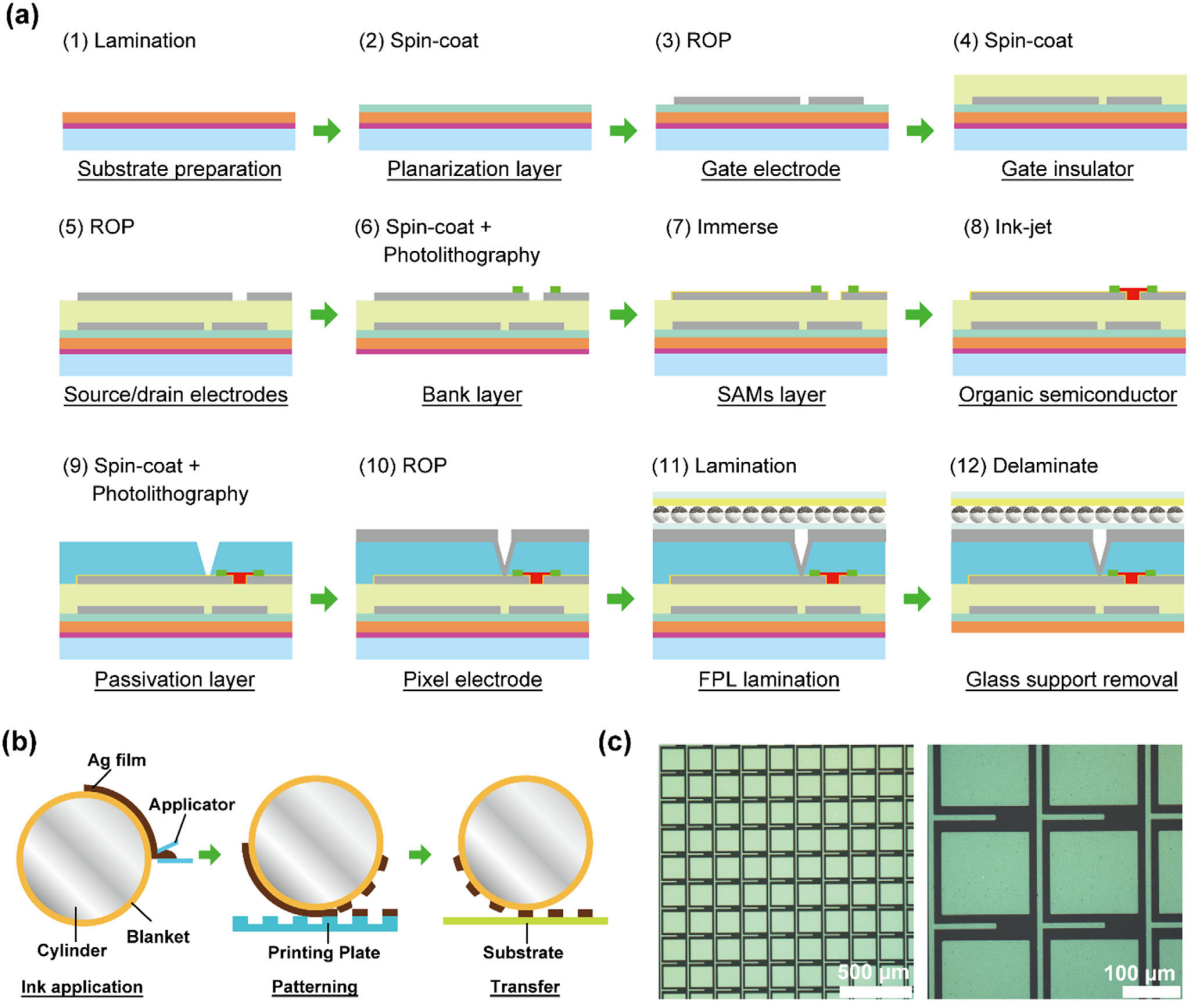
a) Process flow of the printed OTFT backplane on a PEN film temporarily laminated to a glass carrier. Each step specifies the patterning method: reverse‐offset printing (ROP) for the Ag gate/source–drain/pixel electrodes, inkjet for the organic semiconductor, and UV photopatterning for the dielectric, bank, and passivation layers. This is followed by lamination of the electrophoretic layer. The steps are conducted under ambient pressure with a maximum temperature of 160 °C, ensuring compatibility with heat‐sensitive PEN substrates. b) Schematic of ROP. An Ag nanoparticle ink is coated onto a PDMS blanket, selectively eliminated by contact with a relief printing plate, and subsequently transferred to the substrate to form high‐resolution metal patterns. c) Representative optical micrographs of ROP‐formed Ag electrode patterns. The backplane employs a minimum line width of 10 µm and spacing of 15 µm to ensure robust multilayer alignment. For comparison, 5 µm/5 µm patterns employed in the OLED backplane are shown in Figure S5 (Supporting Information).

The organic transistor backplane was fabricated using solution‐based printing processes conducted under ambient pressure with a maximum process temperature of 160 °C. A flowchart of the fabrication process is shown in **Figure** **1**a, whereas detailed process parameters are described in the Experimental Section.

All electrode layers—including gate, source/drain, and pixel electrodes—were fabricated using ROP,^[^ ^33^, ^34^, ^35^, ^36^, ^37^, ^38^ ^]^ a high‐resolution printing technique. The ROP process comprises three steps (Figure 1b): i) deposition of a uniform Ag‐nanoparticle thin film onto a polydimethylsiloxane (PDMS)‐based elastomeric blanket using a glass slit‐coater; ii) selective removal of unwanted regions by contacting the semi‐dried film with a patterned printing plate; and iii) transfer of the remaining patterned film from the blanket onto the substrate, resulting in the final electrode pattern. By suppressing ink flow in the semi‐dry state, ROP enables the fabrication of micrometer‐scale patterns with excellent film‐thickness uniformity and reproducibility. The resulting thin printed layers facilitate the stacking of multilayer electrodes without interference.

Representative optical micrographs of ROP‐formed Ag electrode patterns are shown in Figure 1c. The backplane reported here employs a minimum line width of 10 µm and a minimum spacing of 15 µm, demonstrating both high resolution and excellent linearity.

Utilizing ROP for all electrode layers, we developed a printed, high‐resolution backplane incorporating photopatterned dielectric, bank, and passivation layers. This approach enables reliable multilayer integration on flexible PEN.

Although ROP can achieve feature sizes as small as 1–2 µm under optimized conditions,^[^ ^37^, ^38^, ^39^ ^]^ we intentionally selected a 10 µm line width and 15 µm spacing for the e‐paper backplane. This design choice maximizes manufacturing yield and registration robustness on PEN. Attempting finer features would compromise the mechanical durability of the glass relief plate during repeated printing and cleaning cycles and would reduce the overlay margin between ROP‐printed metal layers and photopatterned dielectric, bank, and passivation layers. Additionally, the selected design provides a favorable RC budget for printed Ag electrodes. For comparison, inkjet‐only metallization typically achieves line/space dimensions of ≈30–50 µm line/space,^[^ ^36^ ^]^ resulting in device footprints at least four times larger for equivalent channel widths. In contrast, we employ a finer 5 µm / 5 µm design rule exclusively for the OLED backplane to maximize the emitting aperture (refer to Figure S5, Supporting Information).

To evaluate interfacial cleanliness following ROP, we performed X‐ray photoelectron spectroscopy (XPS) on the as‐printed Ag electrodes. Under our processing conditions, Si‐related signals—Si 2p at ≈99 eV and Si 2s at ≈150 eV—were consistently observed on the as‐printed Ag. This is attributed to trace transfer from the PDMS blanket. Notably, a simple rinse with isopropyl alcohol (IPA) significantly reduced the intensity of these Si peaks and enhanced wettability. Consequently, IPA rinsing was adopted as a standard post‐print step (Figure S1, Supporting Information). Notably, Si transfer is not an inherent feature of ROP; it can vary depending on the formulation and processing of the PDMS blanket, as well as the solvent uptake characteristics of the ink and blanket. For example, Sneck et al. reported no detectable Si when utilizing a different PDMS system^[^ ^37^ ^]^).

Finally, all backplane fabrication steps were performed at ambient pressure and at temperatures not exceeding 160 °C, utilizing additive printing and coating techniques. This approach reduces both energy consumption and equipment complexity compared to conventional vacuum or high‐temperature processes. Furthermore, the high material utilization efficiency and minimal waste generation inherent to printing methods contribute to more environmentally sustainable manufacturing.

### 2.2 Via‐Hole Formation and Interlayer Connection

In multilayer‐printed backplanes, achieving reliable electrical connections between the lower and upper electrodes necessitates the formation of via‐holes through the dielectric layer.^[^ ^40^, ^41^, ^42^ ^]^ In this study, we investigated the effect of via‐hole sidewall inclination angle on the printability and connectivity of the upper electrode. Furthermore, we compared two types of via‐hole structures, each characterized by different sidewall geometries.

The first via‐hole type was fabricated using a photosensitive polymer dielectric. Following spin coating, square via‐holes with edge lengths of 10, 20, 30, and 40 µm were patterned by photolithographic exposure through a mask, followed by development. This process yielded sidewalls with an inclination angle of 23.8°. Conversely, the second type was fabricated by dry etching using a photoresist mask, resulting in sidewalls with a steeper inclination angle of 34.5°. The inclination angles were measured using a surface profiler (Dektak XT, ULVAC Inc.), and detailed profiles of the via‐hole sidewalls are provided in the Supporting Information (Figure S2, Supporting Information).

**Figure 2:**
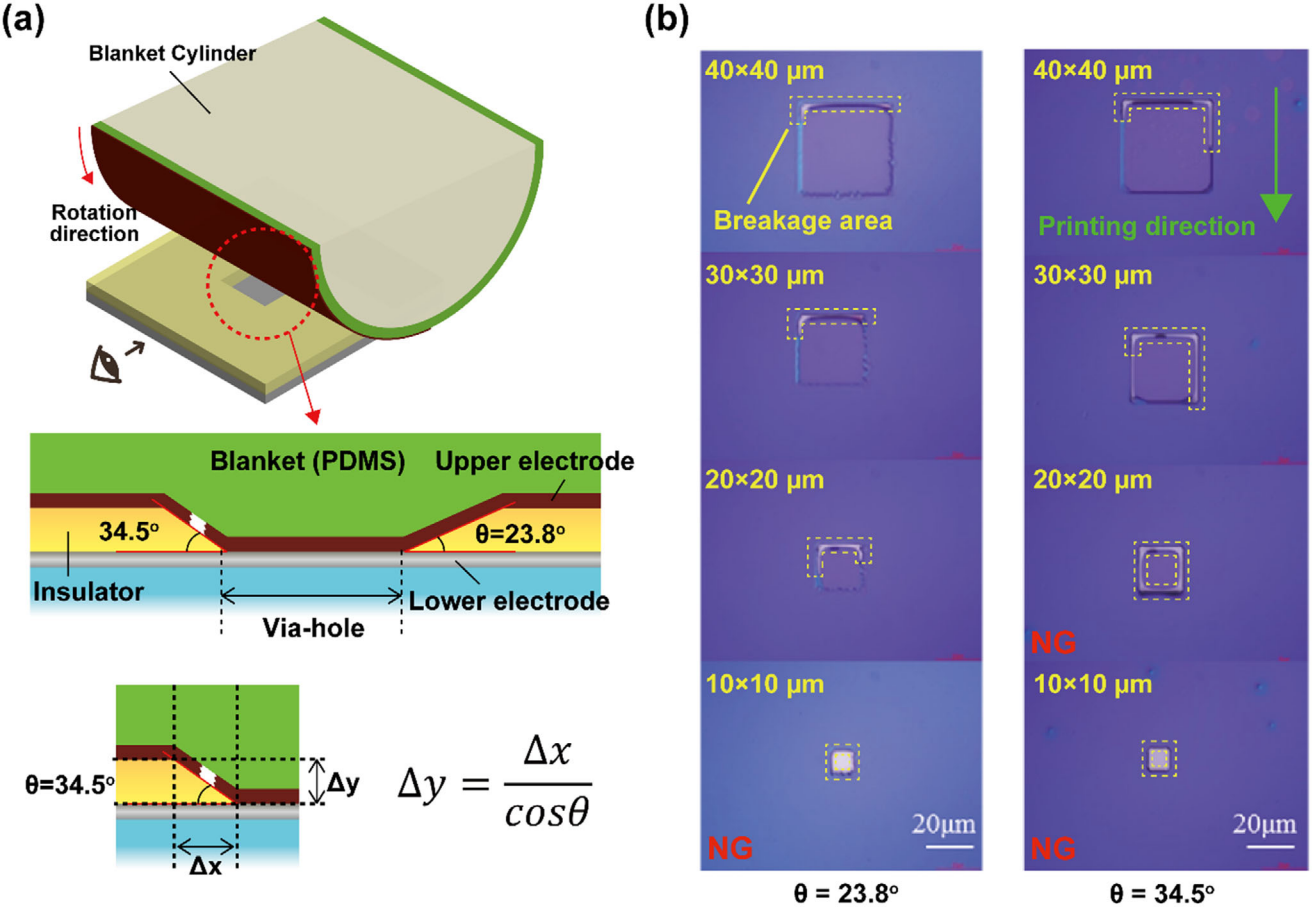
a) Schematic of interlayer connection achieved through reverse‐offset printing. In via‐hole structures, steeper sidewalls increase the risk of silver film rupture and reduce contact with the blanket, resulting in discontinuities within the printed film. b) Electrical continuity results for via‐holes with sidewall angles of 23.8° and 34.5°. The shallower angle (23.8°) supports a reliable connection from 20 to 40 µm, whereas the steeper angle (34.5°) only supports a connection from 30 to 40 µm. The green arrow indicates the printing direction, whereas the yellow dashed box highlights a breakage area in the Ag film where discontinuity occurs.

For upper electrode patterning, we employed an ROP process, in which a pre‐formed silver film on an elastomeric (PDMS) blanket was transferred onto the substrate. As the via‐hole size decreased, the ≈450 nm step height of the insulating layer hindered adequate contact between the blanket and bottom electrode, rendering the formation of reliable interconnections complex (**Figure** **2**a). Moreover, the sidewall inclination angle (θ) emerged as a critical parameter, significantly impacting the continuity of the printed metal film across the via.

In Figure 2a, the x‐axis represents the in‐plane displacement across the via opening, corresponding to the printing or scanning direction on the top surface. In contrast, the variable y in Equation (1) denotes the path length along the sloped sidewall, which does not align with an orthogonal Cartesian axis. Given the sidewall taper angle θ, the required path length is expressed as follows:

(1) $$\Delta y=\frac{\Delta x}{\cos\theta }$$

Therefore, shallower sidewalls (smaller θ) reduce the elongation required for a continuous printed film over the slope.

For example, at θ = 34.5°, Δy is approximately equal to 1.21Δx, whereas at θ = 23.8°, Δy is approximately equal to 1.09Δx. This indicates that steeper sidewalls require a longer printing path, increasing the risk of film rupture if the blanket material cannot accommodate the necessary stretch.

During ROP, the semi‐dry Ag film on the PDMS blanket undergoes in‐plane shear along the printing direction owing to unavoidable roll‐to‐plate slip and the viscoelasticity of the blanket. When a via sidewall is parallel to the printing direction, the deformation required to traverse the slope is distributed along the shear direction and over the entire slope length, promoting continuity. Conversely, when the sidewall is perpendicular to the printing direction, the film must rapidly extend across the slope over a much shorter distance. Combined with stress concentration at the corners, this orientation increases the likelihood of thinning or tearing at the edge. This directionality is consistent with ROP design rules, which emphasize ink thickness, printing speed, and roll‐to‐plate synchronization.^[^ ^40^ ^]^

Electrical continuity testing demonstrated that via‐holes with a 23.8° sidewall angle supported reliable interconnection down to 20 µm (Figure 2b). In comparison, those with a 34.5° sidewall achieved interconnection only down to 30 µm. These results indicate that shallower sidewall angles facilitate better connectivity in smaller vias.

Observed yield (20 µm × 20 µm). Under the fixed printing conditions employed in this study, the electrical continuity yield was ≥ 90% for 23.8° sidewalls but dropped to ≤ 10% for 34.5° sidewalls. This stark contrast underscores the critical role of shallower taper angles in ROP‐based interlayer connections.

Note on via geometry: While circular vias have been explored in previous studies,^[^ ^40^ ^]^ our controlled taper angle study employed square vias (10–40 µm) to systematically achieve sidewall angles of 23.8° (photopatternable insulator) and 34.5° (dry‐etched). However, in the actual backplane layout, we utilize circular vias to minimize stress concentrations at corners and to reduce directional sensitivity.

Our results underscore the critical importance of via‐hole dimensions, sidewall angle, and the orientation of the sidewall relative to the printing direction in establishing robust interlayer connections. For the fabrication of reliable fine‐pitch printed interconnects, optimization of via geometry and the utilization of highly deformable blanket materials are essential.

### 2.3 Structure and Electrical Characterization of Printed OTFTs

**Figure 3:**
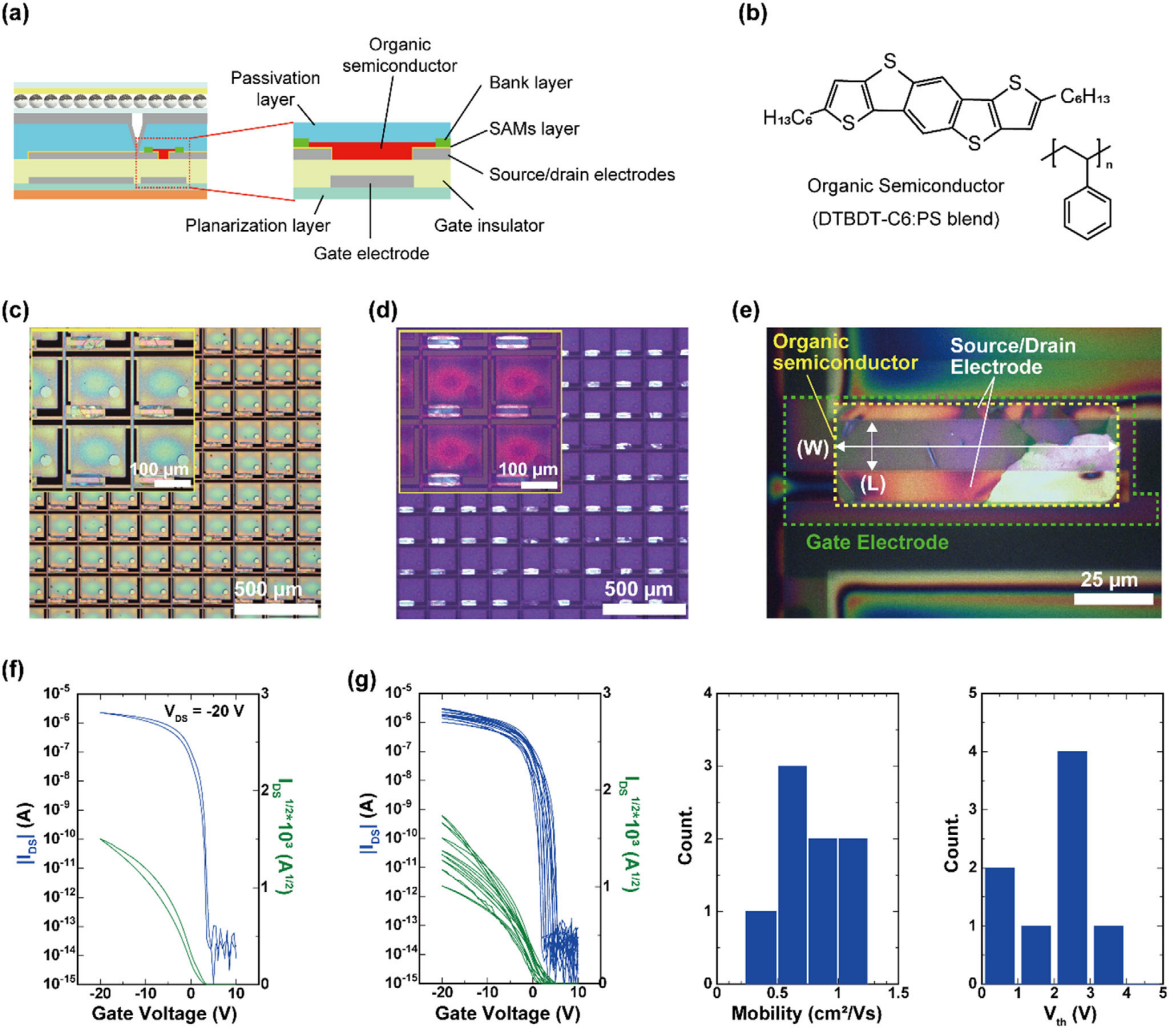
a) Schematic of the printed OTFT with a BGBC structure. b) Chemical structures of DTBDT‐C6 and polystyrene (PS) employed in the semiconductor blend. c) Optical micrograph of the fabricated pixel region. Circular via apertures are used in the pixel design. The sidewall taper angle is approximately equal to 24° (measured by Dektak TX). d) Cross‐polarized optical micrograph of the DTBDT‐C6/PS film within the bank, demonstrating a polycrystalline texture. e) Magnified optical micrograph of a single OTFT. The channel region is delineated (dashed outline). The source (S), drain (D), and gate (G) electrodes, as well as L and W, are indicated. f) Electrical characteristics of a representative OTFT (transfer curve at V_DS_ = −20 V). g) Statistical distribution of field‐effect mobility for n = *8* OTFTs fabricated under identical conditions, indicating device‐to‐device variability.

All transistors investigated in this study were fabricated following the procedure outlined in Section 2.1, utilizing a BGBC structure (**Figure** **3**a). Both the gate and source/drain electrodes were patterned using the ROP technique.

A self‐assembled monolayer treatment using 2,3,4,5,6‐pentafluorobenzenethiol (PFBT) was applied to the source/drain electrodes to minimize contact resistance at the electrode–organic semiconductor interface. Specifically, the electrodes were immersed in a 30 mm ethanol solution of PFBT for 5 min.

The organic semiconductor dithieno[2,3‐d;2′,3′‐d’]benzo[1,2‐b;4,5‐b’]dithiophene (DTBDT‐C6) was employed at a concentration of 0.8 wt.% in mesitylene. To enhance inkjet wettability and facilitate controlled crystallization, 0.1 wt.% polystyrene (PS) was incorporated as an additive. The chemical structures of DTBDT‐C6 and PS are shown in Figure 3b. The DTBDT‐C6/PS blend ink was inkjet‐printed into the bank‐defined channel regions (DMP‐3000, FUJIFILM Dimatix), followed by baking at 90 °C for 10 min to remove residual solvent. Subsequently, a protective dielectric layer was deposited to enhance device stability against ambient oxygen and moisture.

An optical micrograph of the pixel region and a cross‐polarized optical micrograph of the DTBDT‐C6/PS film (both with numeric scale bars) are shown in Figure 3c,d. In the pixel design, the interlayer via apertures are circular, with sidewalls demonstrating a taper angle of ≈24° (measured by Dektak TX). This circular geometry helps mitigate corner‐induced defects and minimizes sensitivity to the printing direction, thereby enhancing metal continuity at the via edge. The specific impact of the sidewall taper angle on interlayer connectivity is discussed in detail in Section 2.2.

To facilitate accurate identification of the channel area, a magnified optical micrograph of a single OTFT is shown in Figure 3e. In this image, the channel region is clearly delineated with a dashed outline, and the source (S), drain (D), and gate (G) electrodes are labeled. The channel length L and width W are also indicated for reference. The DTBDT‐C6/PS film within the bank demonstrates a polycrystalline morphology, consistent with previous findings.^[^ ^43^, ^44^ ^]^

The printed OTFTs demonstrated excellent electrical performance under ambient conditions at room temperature. The devices featured a channel length and width of 15 and 90 µm, respectively. A representative transfer characteristic, measured at V_DS_ = −20 V, is shown in Figure 3f. The transistor displayed an average field‐effect mobility of 1.12 cm^2^ Vs^−1^, a threshold voltage (V_th_) of ≈ −2.3 V, and an ON/OFF current ratio exceeding 10^8^. To avoid confusion arising from range‐setting artifacts, Figure 3f displays only the I_DS_, which is exclusively shown in Figure 3f; the on/off ratio is defined as I_DS_(on)/I_DS_(off) and does not involve gate current. For transparency, the original dataset—including the _IGS_ acquired with a fixed 10 µA gate‐current range—is provided in Figure S7 (Supporting Information). This dataset clarifies the elevated apparent I_GS_ baseline (≈10^−10^ A) despite the much lower intrinsic leakage.

To evaluate the reproducibility of the printing process and uniformity between devices, we characterized the electrical performance of eight transistors fabricated under identical conditions. The summary of the mobility distribution for n = *8* OTFTs fabricated under identical conditions is shown in Figure 3g. The devices demonstrated an average maximum drain current (I_DS_, max) of 1.975 µA (standard deviation of 0.642), an average field‐effect mobility of 0.798 cm^2^ V·s^−1^ (standard deviation of 0.256), and an average threshold voltage (V_th_) of −1.99 V (standard deviation of 1.03).

Uniformity considerations: In the current two‐tone electrophoretic display, each pixel TFT primarily serves as a voltage switch or holder. Therefore, device‐to‐device variations have a limited visual impact and can be effectively compensated for through drive waveforms and software adjustments. However, applications, such as OLEDs and multicolor or grayscale electrophoretic displays, are significantly more sensitive to variations in current and threshold voltage, necessitating tighter device uniformity. The observed spread is primarily attributed to the polycrystalline microstructure of the DTBDT‐C6/PS semiconductor and its interaction with short channel lengths. This device‐to‐device variation correlates with the polycrystalline microstructure of DTBDT‐C6/PS in the channel regions, as shown in the cross‐polarized panel in Figure S6 (Supporting Information). To mitigate this, we are actively exploring the use of polymer semiconductors and optimizing both the printing and thermal treatments to reduce grain‐related variability and narrow parameter distributions.

Although the observed variations—particularly in mobility and threshold voltage—are somewhat greater than those found in highly uniform devices, they remain within acceptable limits given the small channel dimensions and inherent variability associated with organic semiconductor crystallinity and the printing process. Notably, for applications such as electrophoretic displays, where transistors primarily serve as switching elements to apply voltages to individual pixels, this level of variability has a negligible effect on overall display performance. Any residual variation can be effectively managed through circuit design or software‐based control. Therefore, the printed transistors developed in this study are well‐suited for use in e‐paper display technologies.

### 2.4 Demonstration of E‐Paper and OLED Display Driving

In this study, we demonstrated the operation of both electrophoretic (e‐paper) and OLED displays utilizing an active‐matrix backplane fabricated entirely through printing and solution‐based processes under ambient pressure and low temperatures (≤ 160 °C).

**Figure 4:**
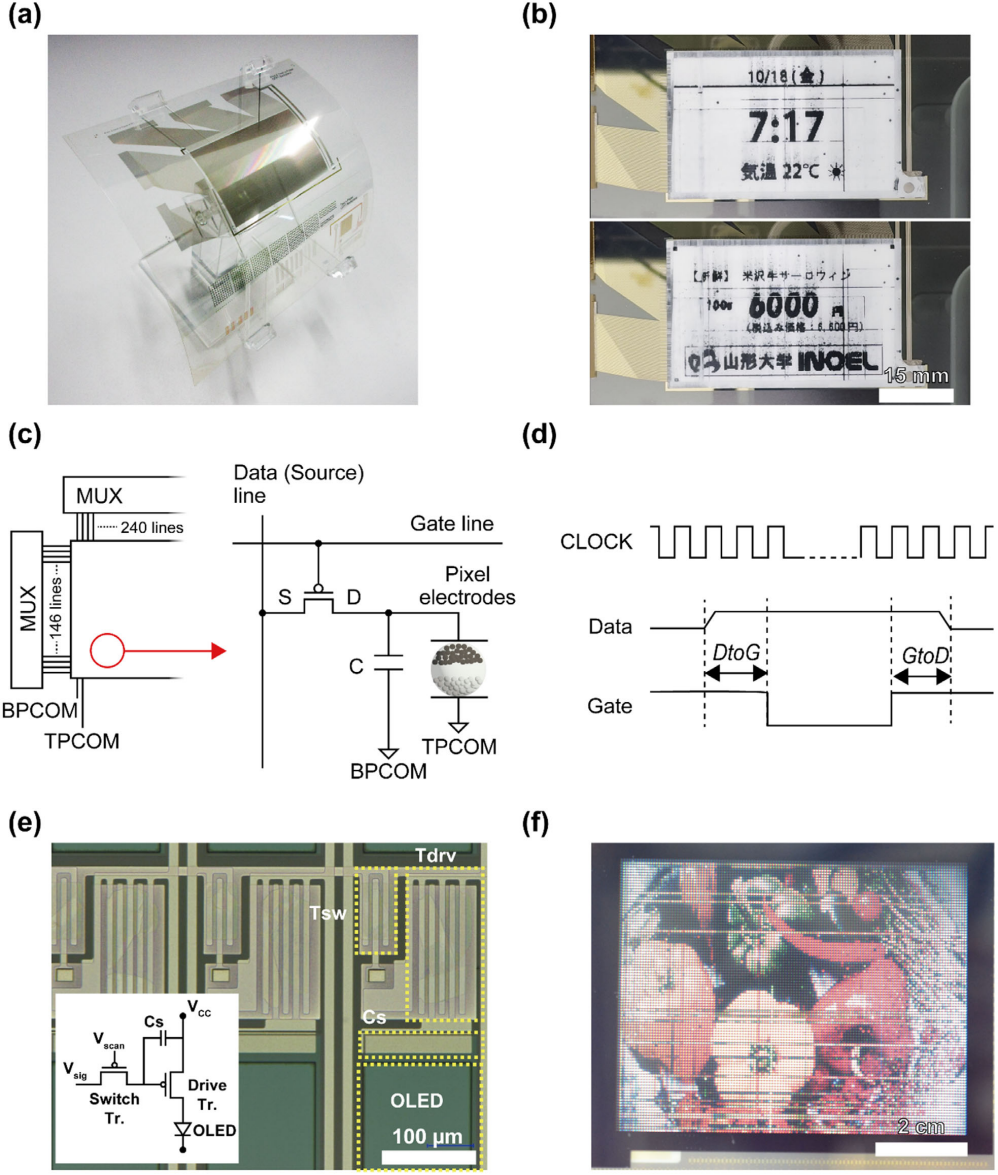
a) Photographs of the printed backplane prior to lamination. b) Photograph of the backplane laminated with the electrophoretic film (pattern display shown). The backplane has dimensions of 27.7 mm × 45.6 mm (2.1 in.) and features a resolution of 240 × 146 pixels (133.7 dpi). c) Schematic of the e‐paper driving system (left) and 1T–1C pixel circuit (right) employed to write and hold the pixel voltage. d) Clock, data‐line, and gate‐line waveforms utilized for display driving. A delay of 10 clock cycles (40 µs at 250 kHz) is applied between the data and gate signals in both directions (DtoG and GtoD) to ensure stable pixel switching. e) OLED pixel micrograph with the inset circuit diagram displaying a 2T–1C architecture (switching TFT Tsw, driving TFT Tdrv, storage capacitor Cs, supply rail V_CC_, and OLED). The numerical scale bar is shown in the panel. f) Photograph of the operating OLED display (refer to Movie S2, Supporting Information as well). The OLED stack and fabrication process flow are shown in Figures S4 and S3 (Supporting Information), respectively. The backplane has dimensions of 50.7 mm × 64.9 mm (3.2 in.) and features a resolution of 128 × 3 × 100 pixels (50 ppi).

Photographs of the printed backplane prior to lamination are shown in **Figure** **4**a. The backplane achieves a resolution of 240 × 146 pixels (133.7 dpi) within an area of 27.7 × 45.6 mm (≈2.1 inches), showcasing the potential of printing and solution processing for high‐density active‐matrix configurations. To realize a functional e‐paper display, an electrophoretic front panel (Aurora, E Ink Holdings) was laminated onto the backplane and electrically integrated, as shown in Figure 4b. The system‐level driving circuit and 1T–1C pixel architecture—comprising a single switching TFT for voltage writing and a storage capacitor for charge retention—are shown in Figure 4c.

The waveform diagram is shown in Figure 4d, which depicts the clock signal, data‐line voltage, and gate‐line voltage during display operation. Timing offsets between these signals, defined as the delay from data to gate (DtoG) and delay from gate to data (GtoD), were both set to 10 clock cycles. The driving IC operates with a 100 MHz master clock, which is divided by 400 to generate a 250 kHz timing clock. Therefore, 10 clocks corresponded to 40 µs. During device operation, the gate‐line voltages were ±30 V (High: +30 V, Low: −30 V), whereas the common electrodes BPCOM and TPCOM were fixed at 0 V. The data‐line voltages were +14.88 V (black) and −20 V (white), enabling polarity‐controlled particle migration across the FPL electrodes for stable black‐and‐white transitions.

To achieve high image quality and minimize residual charges, we implemented a two‐phase driving sequence comprising both refresh and image‐writing stages. During the refresh phase, the display was alternately cycled between fully white and fully black images (such as white → black → white) for 15 cycles, effectively erasing any residual images. This was followed by 15 cycles of the desired image pattern, with each pattern maintained for 15 clock intervals to stabilize the visual output.

Using this approach, we successfully displayed patterned images on the electrophoretic panel, leveraging fully printed transistors. This not only confirmed the proper functionality of the transistors but also demonstrated effective charge retention (Figure 4a; Movie S1, Supporting Information).

For the OLED backplane, we employed a 2T–1C pixel architecture. The display consisted of 128 × 3 × 100 pixels (50 ppi) within an active area of 50.7 × 64.9 mm (≈3.2 inches). In this design, a switching TFT (Tsw) writes the data voltage onto a storage capacitor (Cs), which is connected to the gate of the driving TFT (Tdrv). The driving TFT then sources current from the V_CC_ rail to the OLED emitter. A representative micrograph of an OLED pixel, along with an inset circuit diagram, is shown in Figure 4e, whereas a photograph of the operating OLED display is shown in Figure 4f (refer to Movie S2, Supporting Information). The OLED fabrication process flow and device stack are shown in Figures S3 and S4 (Supporting Information), respectively. To optimize the local electrode pattern in the OLED backplane, we selected a 5 µm/5 µm line/space configuration. This design maximized the emitting aperture and accommodated higher drive currents, albeit at the expense of mechanical robustness compared with the more conventional 10 µm/15 µm plate (Figure S5, Supporting Information).

The demonstrator devices displayed a limited number of dead pixels and line defects, which were primarily attributed to particle contamination introduced during manual substrate handling and lamination, even under cleanroom conditions. In the current prototype workflow, several inter‐stack transfers were performed manually, making operator‐borne particles the predominant source of observed failures, such as open vias, interconnects, and occasional shorts. Comprehensive yield optimization—including process automation (enclosed, cassette‐to‐cassette transfer), enhanced enclosure of ROP/lamination modules, and pre‐bond surface cleaning (such as ionized air or tacky‐roller removal)—remained beyond the scope of this proof‐of‐concept study but will be a focus in future scale‐up efforts.

The printed active‐matrix backplane developed in this study demonstrated sufficient functionality and versatility to drive multiple types of display elements, including electrophoretic and OLED devices. These results underscore the significant potential for future applications in printed electronics.

## 3 Conclusion

In this study, we developed a high‐resolution active‐matrix organic transistor backplane, fabricated entirely through printing techniques. We demonstrated its robust capability to drive both e‐paper and OLED displays.

Employing ROP, we achieved uniform, high‐resolution electrode patterns with a minimum line width of 10 µm and line spacing of 15 µm—dimensions well‐suited for multilayer integration in display backplanes. The printed OTFTs demonstrated excellent electrical performance under ambient conditions, achieving a mobility of 1.12 cm^2^ Vs^−1^ and an on/off ratio exceeding 10⁸. These characteristics satisfy the requirements for pixel switching in e‐paper displays.

To ensure reliable multilayer interconnections, we examined the impact of via‐hole sidewall geometry. Our findings revealed that via‐holes with shallower angles (23.8°), formed using photopatternable insulators, provided significantly more reliable electrical connectivity compared with those of the steeper angles (34.5°) formed by dry etching. This insight highlights the advantages of fully printed processes for complex multilayer structures.

By integrating the printed backplane with a commercial electrophoretic layer, we achieved stable and patternable e‐paper display operation. Furthermore, we successfully extended the same printing processes to OLED displays, further demonstrating the adaptability and broad applicability of our printed backplane platform across diverse display technologies.

Importantly, XPS analyses revealed the presence of Si‐related signals on as‐printed Ag films transferred from the PDMS blanket. Notably, these signals were significantly diminished following a simple IPA rinse. Collectively, these findings demonstrate that Si transfer during ROP is not universal but can vary with PDMS blanket formulation and processing, ink–blanket solvent uptake, and the operational time window, such as the coating‐to‐contact delay. This underscores the importance of co‐optimizing both blanket chemistry and process timing to achieve optimal interfacial cleanliness.

These results highlight printed backplane technology as a scalable and versatile platform for next‐generation flexible displays. In the future, further advancements in fine‐pattern ROP, the development of higher‐mobility printable semiconductors, and the integration of self‐aligned processing techniques will be critical for enabling larger display areas and higher pixel densities. Finally, conducting all fabrication steps at ambient pressure and low temperature (≤ 160 °C), while utilizing additive printing and coating methods, can substantially reduce both energy consumption and material waste. This approach offers a compelling pathway toward environmentally sustainable manufacturing of flexible electronics.

## 4 Experimental Section

### Fabrication Process of E‐paper Display—Fabrication Process

Initially, a 125 µm‐thick polyethylene naphthalate (PEN) film (Q65HA, Toyobo Co., Ltd.) was temporarily laminated onto a glass carrier substrate using double‐sided adhesive tape (CS2325NA2, Nitta Corp.) to ensure mechanical stability and flatness. The PEN surface was then planarized, and its surface free energy was tailored by spin‐coating a 300 nm‐thick cross‐linked poly(4‐vinylphenol) (PVP) insulating layer, followed by thermal curing at 150 °C for 1 h.

*Post‐print Surface Cleaning (General Rule)*: Immediately after each ROP step (gate, source/drain, and pixel), the printed Ag electrodes were rinsed with IPA (IPA, ≥ 99.5%) and subsequently dried with nitrogen prior to the deposition of the next layer. This straightforward cleaning procedure reliably suppressed silicon‐related XPS signals, enhanced interfacial wettability, and improved the overall performance of the OTFT devices (Figure S1, Supporting Information).

Subsequently, the gate electrode was patterned using ROP with a 19 wt.% Ag nanoparticle ink (ULVAC, Inc.), yielding a uniform 110 nm‐thick layer. Post‐baking was performed at 160 °C for 1 h.

For the gate dielectric, a negative‐type photosensitive styrene‐based polymer (DC100E, Tosoh Corp.) was spin‐coated to a thickness of 480 nm, photopatterned using a UV dose of 200 mJ cm^−^^2^, and developed in xylene to open contact holes and eliminate excess material.

Subsequently, source/drain electrodes were fabricated by ROP using a 15 wt.% Ag nanoparticle ink (ULVAC, Inc.), achieving a final thickness of 80 nm. These electrodes were then thermally annealed at 160 °C for 1 h (then IPA rinsed as previously elucidated). The semiconductor channel region was defined by forming a bank layer. This was accomplished by spin‐coating a negative‐type photosensitive fluoropolymer (FD400, Tosoh Corp.) to a thickness of 250 nm, followed by UV exposure at 250 mJ cm^−2^ and subsequent development using acetone.

Prior to semiconductor deposition, the device surface was treated with 30 mm pentafluorobenzenethiol (PFBT) for 5 min to enhance interfacial wettability. The organic semiconductor layer was then deposited within the bank structure by inkjet printing a blend of dithieno[2,3‐d;2′,3′‐d’]benzo[1,2‐b;4,5‐b’]dithiophene (DTBDT‐C6, 0.8 wt.%) and polystyrene (0.1 wt.%). This layer was subsequently baked at 90 °C for 10 min.

Next, a photosensitive insulating film (FD400, Tosoh Corp.) was spin‐coated to a thickness of 0.45 µm, serving as a protective layer. The film was patterned by UV exposure (900 mJ cm^−^^2^) through a photomask and developed using fluorinated solvents (AE‐3000, AGC Inc.; SF10, Mitsui‐Chemours Fluoroproducts Co., Ltd.), which selectively removed the film from contact hole regions and other undesired areas.

Subsequently, a 15 wt.% Ag nanoparticle ink (ULVAC, Inc.) was employed to print the pixel electrode, achieving a thickness of 110 nm through the ROP. The printed electrodes were then annealed at 160 °C for 1 h.

To establish electrical contact between the TPCOM (common electrode) of the electrophoretic panel and backplane, a commercial Ag paste was manually dispensed and evenly spread across the designated overlap area (no photopatterning). Care was taken to prevent short circuits to adjacent lines during this process. Following the paste application, the FPL (E Ink Aurora) was laminated onto the backplane according to the recommended procedure of the panel vendor. The assembled module was then visually inspected to ensure uniform contact and the absence of air gaps within the active area. Finally, the carrier glass substrate was peeled off, resulting in a flexible, fully printed OTFT backplane.

Notably, the entire fabrication process was conducted at temperatures not exceeding 160 °C, which enabled direct fabrication on thermally sensitive PEN substrates.

### Fabrication Process of E‐paper Display—Interconnection and Fan‐Out

Backplane fan‐out pads (Ag, opened through the passivation) were connected to a flexible printed circuit (FPC) by anisotropic conductive film (ACF) thermo‐compression bonding. Precise alignment was accomplished using a pad and fiducial marks observed under a stereomicroscope, followed by bonding under the standard conditions specified by the ACF supplier. The FPC was subsequently interfaced with a dedicated driver board for e‐paper or OLED operation and measurement. Prior to the final display assembly, the continuity of all input/output lines was validated by probing the FPC header.

### Materials—Silver Nanoparticle Inks

All printed metal layers were fabricated using Ag nanoparticle inks (L‐Ag RP0, ULVAC, Inc.). The gate electrode ink contained 19 wt.% solids, whereas the source/drain and pixel electrode inks contained 15 wt.% solids. According to the safety data sheet, the inks comprise Ag nanoparticles with a mean particle size of ≈3 nm, dispersed in a mixed solvent of octane and 2‐ethylhexane, along with proprietary additives. The viscosity of the ink was ≈5 mPa·s at 25 °C.

### Materials—PDMS Blanket

A two‐part PDMS (X‐32‐3279‐A/B, Shin‐Etsu Chemical) was prepared according to the manufacturer's instructions and cured at 150 °C for 30 min. The blanket thickness was 0.2 mm on a 0.25 mm PET support. Key mechanical and surface properties included a Shore A hardness of 59, low‐molecular‐weight siloxane‐cut, antistatic characteristics, surface free energy of 14.9 mJ m^−2^, and pot life exceeding 24 h.

### Reverse Offset Printing

*Control of the Semi‐Dry State on the PDMS Blanket*: The Ag film coated on the PDMS blanket was allowed to partially dry for ≈1 s (coating‐to‐contact delay, t_wait_≈1 s) under ambient room‐temperature cleanroom conditions before contacting the printing plate. At this interval, the film demonstrated a mirror‐like silver surface, which facilitated the reliable transfer of the non‐patterned regions by the plate. Prolonged waiting times resulted in transfer defects. Throughout this study, this time‐based operational criterion was strictly maintained.

*Process Speed and Timing*: To ensure consistent timing between coating and transfer, the linear speed was set to 20 mm·s^−1^ for both the coating and plate transfer steps. The coating‐to‐plate contact delay was fixed at ≈1 s to reproducibly establish the semi‐dry state on the PDMS blanket. Under these controlled conditions, the via‐hole connectivity trends discussed in Section 2.2 primarily reflect variations in via geometry, specifically the sidewall taper angle and its orientation relative to the printing direction.

*Via Aperture Shape and Profiling*: For the pixel layout, circular via apertures were employed to suppress corner‐related defects and reduce sensitivity to the printing direction. The sidewall taper angle was measured to be ≈24°, as determined by Dektak TX profilometry.

### Statistical Analysis

*Sample size (n)*: n denotes the number of independent devices measured under identical conditions. For the OTFT statistics presented in Figure 3 g, n = 8 devices were characterized on the same substrate design.

*Metric Extraction*: Transfer characteristics were recorded at V_DS_ = −20 V with V_GS_ swept from −20 to +10 V. The saturation mobility (µs_at_) was extracted from a linear fit of √|I_DS_| versus V_GS_ in the saturation region, whereas the threshold voltage (V_th_) was determined from the intercept of the same fit. The maximum drain current was defined as the peak I_DS_ observed within the specified V_GS_ sweep at V_DS_ = −20 V. The on/off ratio was calculated as |I_DS_, on|/|I_DS_, off|, using the extrema of the sweep window. All parameter extractions employed a consistent algorithm and fitting window for all devices.

*Data Presentation*: Unless otherwise stated, values were reported as mean ± standard deviation.

*Pre‐processing*: Raw I–V data were examined for instrument artifacts, such as range overflows or obvious probe loss. No additional data transformations were applied beyond the mobility/threshold extraction previously defined.

*Outliers*: Data points were retained unless clear evidence of a measurement artifact was observed, such as open or short circuits resulting from probe miscontact.

### Electrical Measurement Settings

Transfer and output characteristics were acquired using a Keithley 4200A parameter analyzer (Clarius v1.9) under ambient conditions. For the main‐text plots, I_DS_ used LimitedAuto 1 nA to enhance low‐current sensitivity. I_GS_ was omitted from these plots to avoid baseline variations associated with range‐dependent measurements. Gate current was originally acquired using a fixed 10 µA range to ensure measurement speed and stability. These data are presented in Figure S7 (Supporting Information) to document the origin of the elevated apparent I_GS_ baseline (≈10^−10^ A).

## Conflict of Interest

The authors declare no conflict of interest.

## Supporting information



Supporting Information

Supplemental Movie 1

Supplemental Movie 2

## Data Availability

The data that support the findings of this study are available from the corresponding author upon reasonable request.
