# Supplementary material for: Low‐Temperature, Sustainable Manufacturing of Printed OTFT Backplanes for Electrophoretic and OLED Displays
Source: Adv Sci (Weinh). 2025 Sep 30;13(2):e14091. doi: 10.1002/advs.202514091 (PMC12786328; doi:10.1002/advs.202514091)
Supplement: Supplementary file 1 — Supporting Information [file ADVS-13-e14091-s001.docx]

Supporting Information

Low-Temperature, Sustainable Manufacturing of Printed OTFT Backplanes for Electrophoretic and OLED Displays

*Yasunori Takeda*, Miho Abiko, Kaori Watanabe, Ryoko Horie, Yasutaka Nakamura,*

*Junghwi Lee, Shohei Yumino, Shinya Oku, Tomohito Sekine and Makoto Mizukami**

To evaluate the surface cleanliness of Ag electrodes formed by reverse-offset printing (ROP), XPS measurements were conducted on samples both before and after rinsing with isopropyl alcohol (IPA). The XPS spectra of the untreated samples revealed a pronounced Si signal, which is attributed to polydimethylsiloxane (PDMS) residues transferred from the printing blanket. However, this Si peak was significantly diminished following IPA rinsing, demonstrating that surface contaminants introduced during the printing process can be effectively removed through a straightforward rinsing step. These findings underscore the efficacy of IPA rinsing in enhancing interface quality, which is critical for the reliable fabrication of printed transistors.


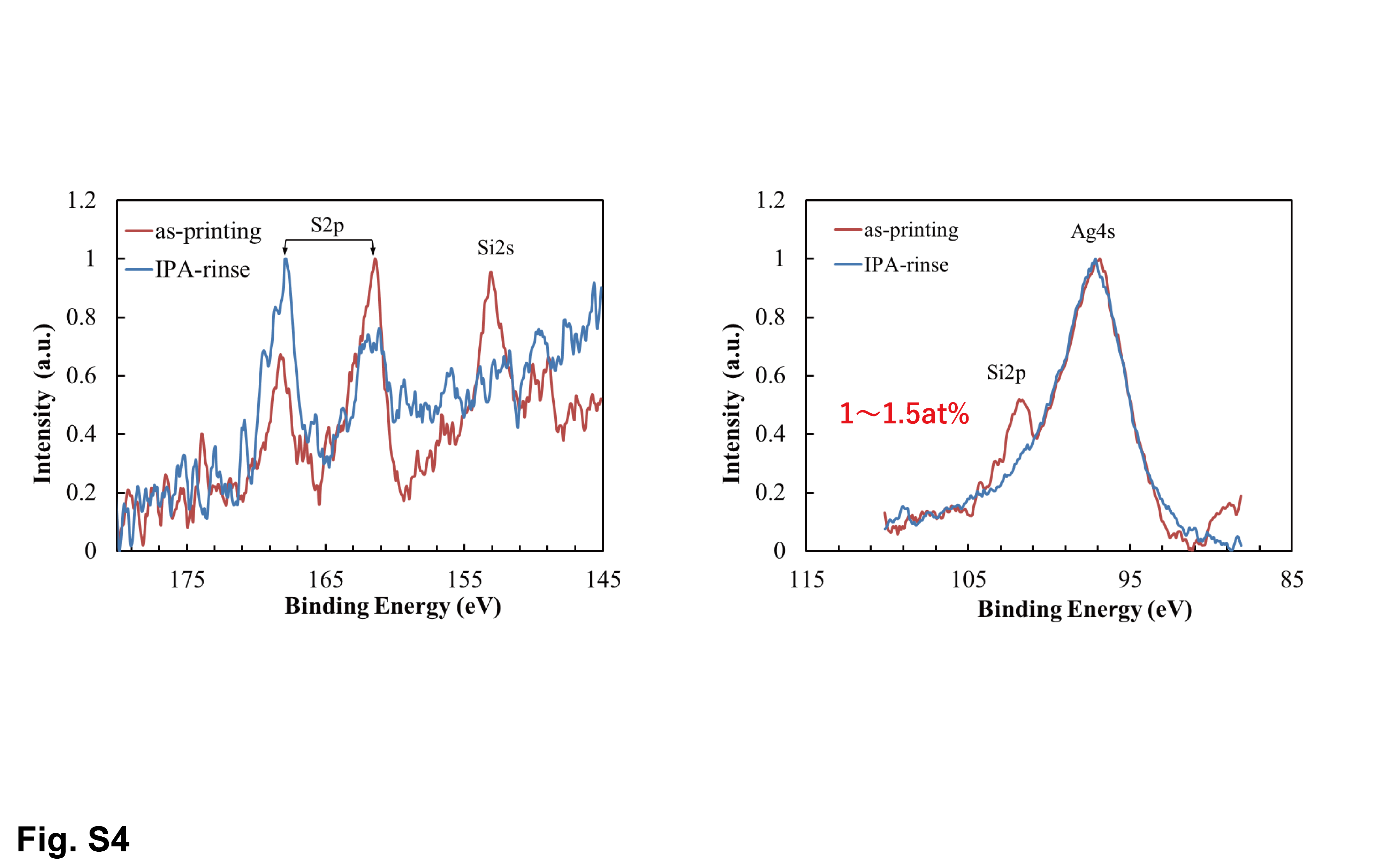


**Figure S1.** XPS spectra of Ag electrode surfaces before and after IPA rinsing. (left) Binding energy range of 145–179 eV, indicating the Si 2s region. (right) Binding energy range of 85–115 eV, displaying the Si 2p region. Si peaks are present in as-printed samples and are significantly reduced after IPA rinsing. This is consistent with the removal of PDMS-derived residues from the ROP blanket.

**
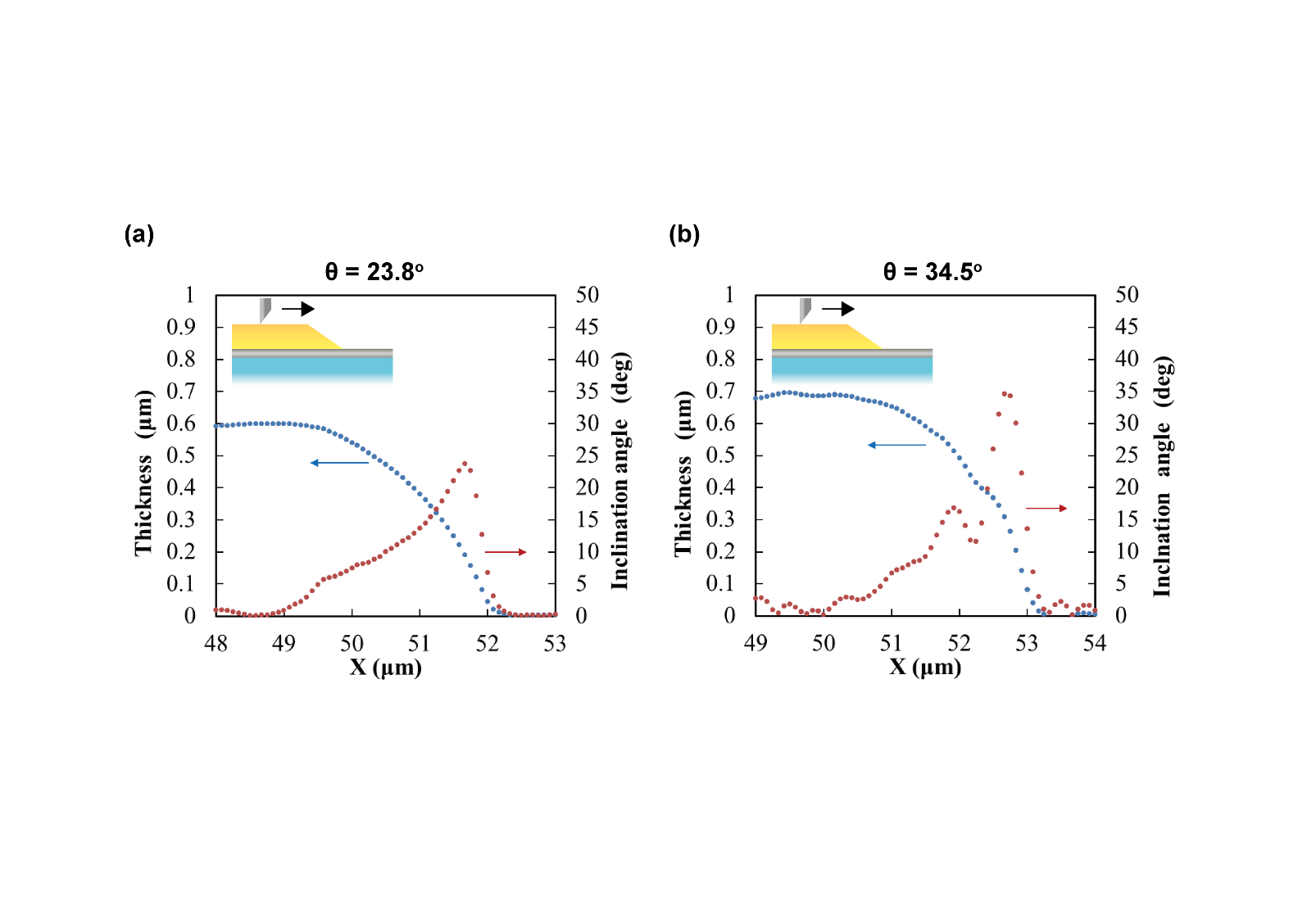
**

**Figure S2.** Cross-sectional profiles of via-holes and corresponding inclination angles measured using a surface profiler (Dektak 8, ULVAC Inc.). (a) Via-hole formed using a photosensitive polymer dielectric (maximum inclination angle: 23.8°). (b) Via-hole formed through reactive ion etching (RIE) (maximum inclination angle: 34.5°).

**Fabrication Process of the Backplane for OLED Display**

A 125 µm-thick polyethylene naphthalate (PEN) film (Q65HA, Toyobo Co., Ltd., distributed by Teijin) was temporarily laminated onto a glass carrier substrate using a double-sided adhesive tape (CS2325NA2, Nitta Corp.). To planarize the PEN surface and control its surface free energy, a 300 nm-thick cross-linked poly(4-vinylphenol) (C-PVP) insulating layer was spin-coated and thermally cured at 150 °C for 1 h. A 320 nm-thick Si₃N₄ barrier layer was deposited by sputtering. An indium zinc oxide layer, 200 nm in thickness, was then sputtered, patterned through photolithography, and etched using an ITO etchant (ITO-07N, Kanto Chemical Co., Inc.). The gate electrode was formed by ROP of a 19 wt% Ag nanoparticle ink (ULVAC, Inc.) to a thickness of 110 nm. This was followed by annealing at 160 °C for 1 h. For the gate dielectric, a 480 nm-thick photosensitive insulating layer (DC100E, Tosoh Corp.) was spin-coated onto the substrate. The layer was then patterned by UV exposure (200 mJ/cm²) through a photomask and developed in xylene to selectively open contact holes and remove undesired regions. Source and drain electrodes were fabricated by ROP with a 15 wt% Ag nanoparticle ink (ULVAC, Inc.), resulting in an 80 nm-thick layer, which was also annealed at 160 °C for 1 hour. A bank layer was subsequently formed by spin-coating a 50 nm-thick photosensitive insulating film (FD400E, Tosoh Corp.), which was photopatterned using UV exposure (100 mJ/cm²) and developed with acetone and IPA. Prior to semiconductor deposition, the surface was treated with a 30 mM solution of pentafluorobenzenethiol in ethanol for 5 min to enhance surface wettability. The organic semiconductor ink, comprising DTBDT-C6 (0.8 wt%) and polystyrene (0.1 wt%) dissolved in mesitylene, was inkjet-printed into the bank-defined regions and baked at 90 °C for 10 min to evaporate residual solvent. A 2.5 µm-thick protective layer was then applied by spin-coating a photosensitive insulating film (FD400, Tosoh Corp.). UV exposure at 300 mJ/cm² was performed using a photomask, followed by development with fluorinated solvents (AE-3000, AGC Inc.; FC-3283, 3M) to define the anode region and eliminate excess film. Subsequently, the substrate underwent a 7-min UV ozone treatment using a UV-1 cleaner. Finally, the OLED functional layers and cathode electrodes were then deposited in a vacuum chamber. Finally, a color filter layer was laminated to complete the OLED display structure.

**
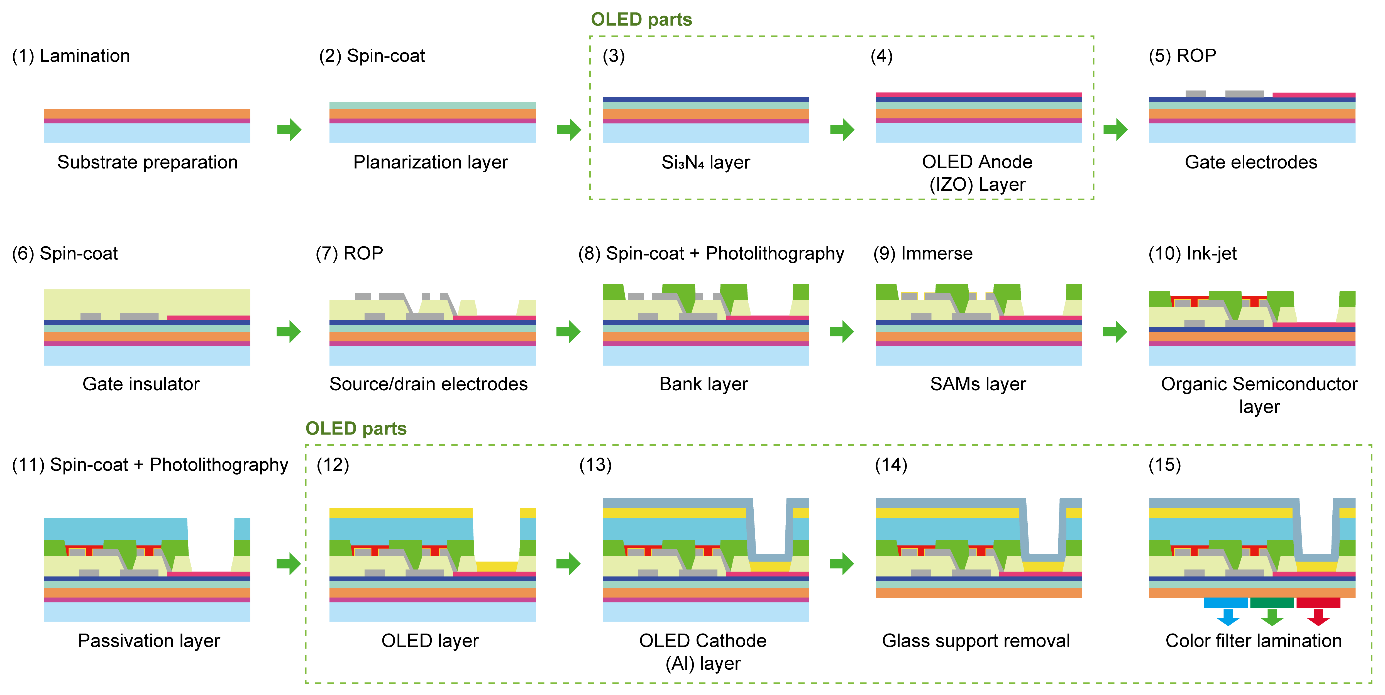
**

**Figure S3**. Process flow for OLED display fabrication utilizing the printed backplane. The key steps include barrier layer deposition, patterning of IZO electrodes, formation of printed electrodes and bank structures, inkjet deposition of organic semiconductors, and lamination of color filters after OLED layer and cathode formation.


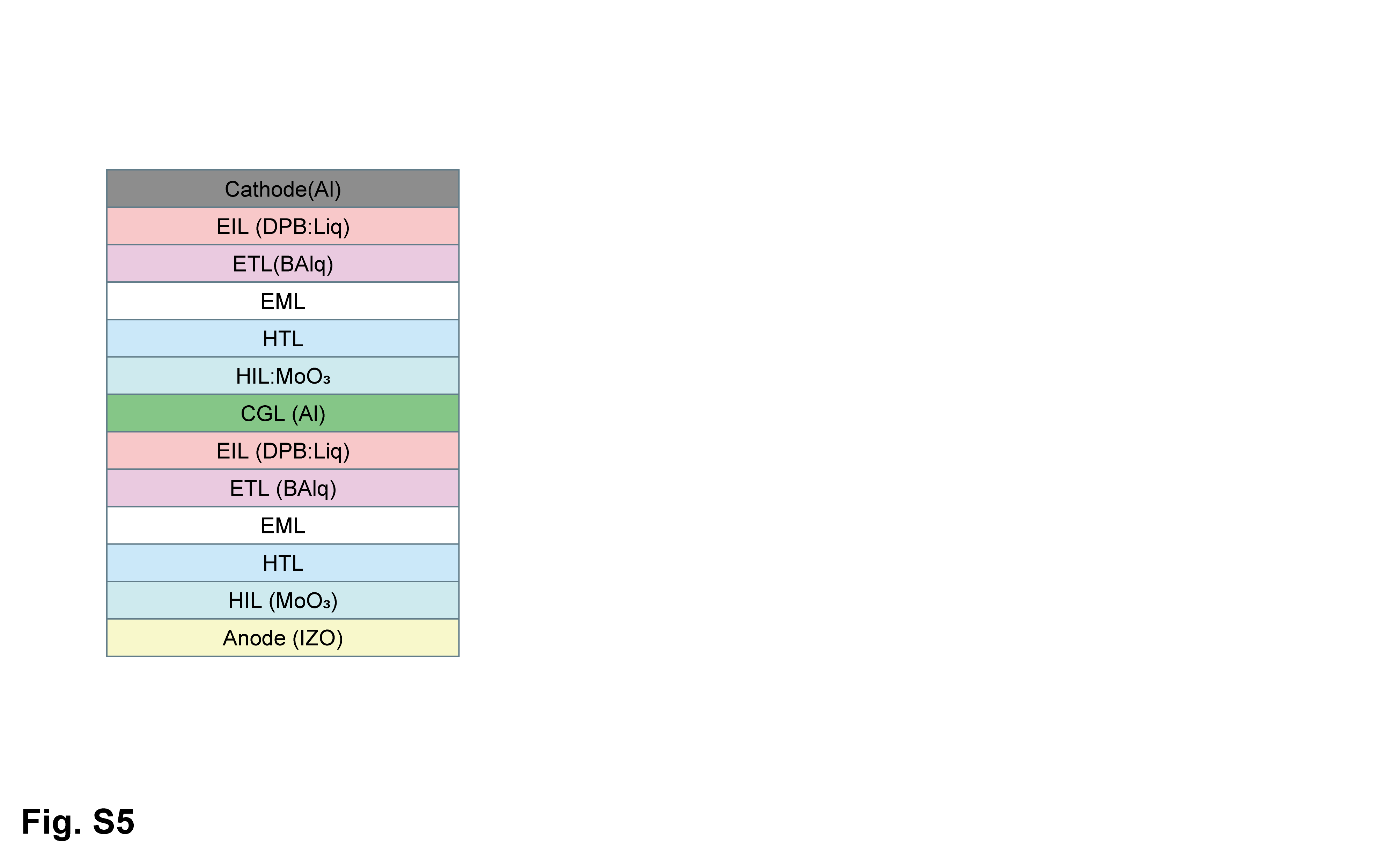


**Figure S4**. Layer structure of the OLED emission stack. The device comprises a multilayer structure including a hole injection layer, hole transport layer, emissive layer, electron transport layer, and electron injection layer, capped with a reflective Al cathode.


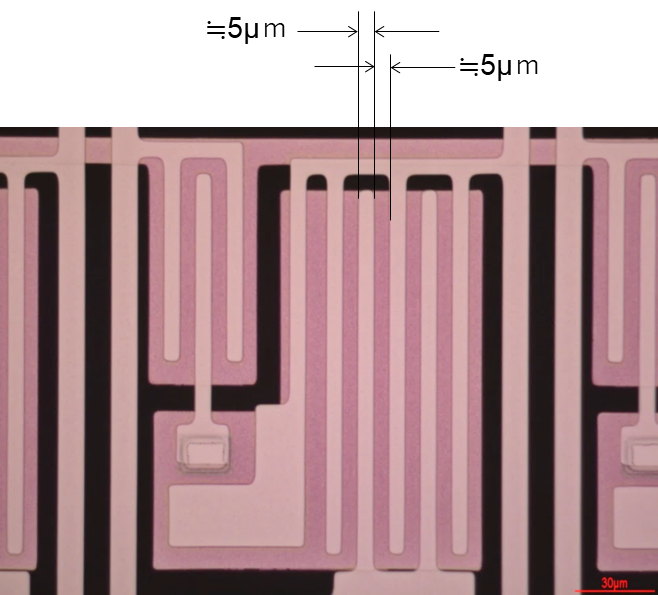


**Figure S5.** Optical micrographs of 5 µm/5 µm Ag line/space electrode patterns for the OLED backplane, fabricated by ROP using the same printing machine described in the main text. These images demonstrate the finer resolution attainable with ROP under the present ink/blanket conditions. For the backplane reported in the main text (e-paper), 10 µm/15 µm was selected to prioritize yield, interlayer overlay alignment, and glass relief-plate durability on the flexible PEN.

**
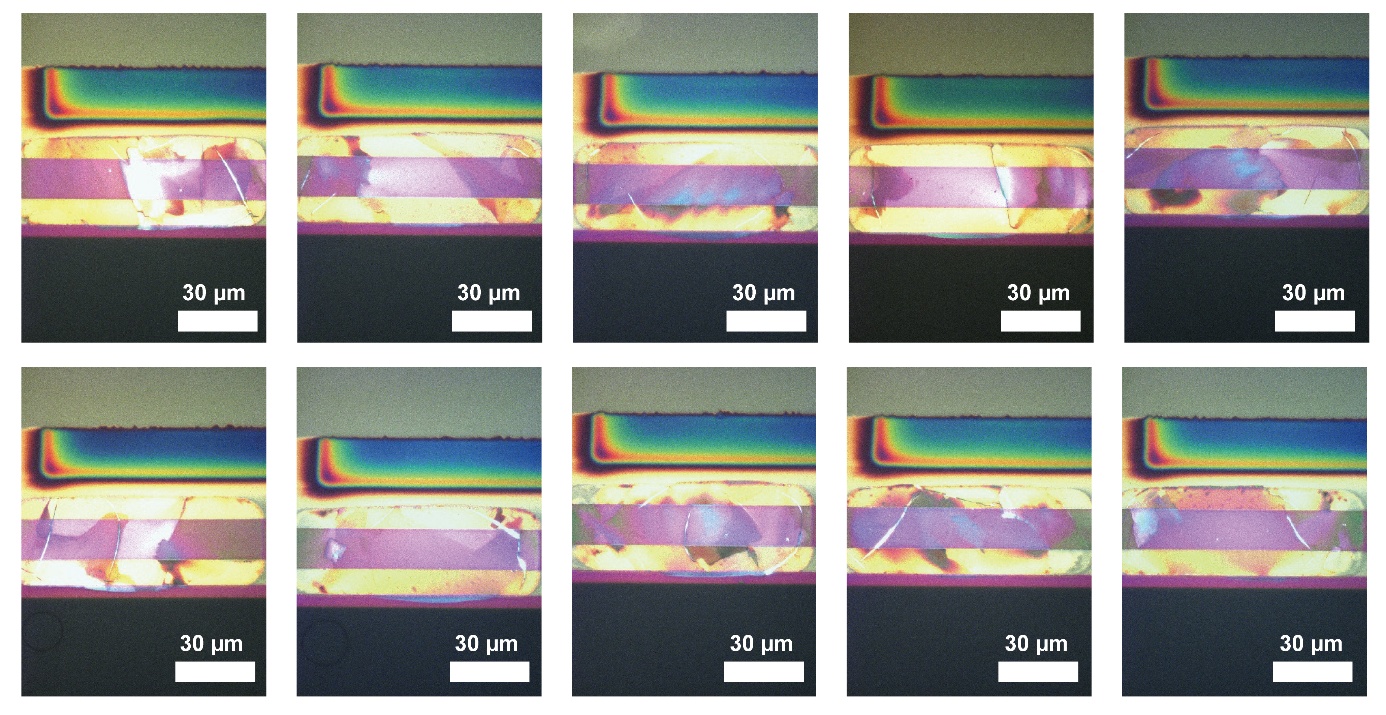
**

**Figure S6.** Cross-polarized optical micrographs of the channel regions of ten OTFTs, each inkjet-printed with DTBDT-C6 under identical process conditions. The images highlight device-to-device variability in crystalline texture (grain size, domain orientation, and boundary density); refer to Section 2.3. Scale bar (all panels): 30 µm.

**
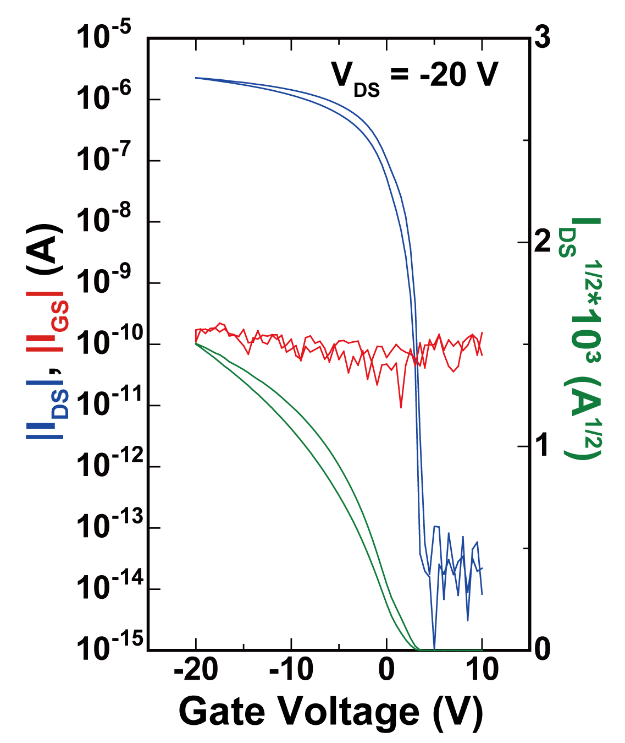
**

**Figure S7**. Representative transfer characteristics displaying both I_DS_ and I_GS_, measured using the original fixed 10 µA gate-current range (Keithley 4200A, Clarius v1.9). The elevated I_GS_ baseline near approximately 10^–10^ A is attributed to the selected measurement range, rather than intrinsic gate leakage. In the main text, the graph I_DS_ exclusively is shown in Figure 2f, and the on/off ratio is defined as I_DS_ (I_DS_(on)/I_DS_(off)).

**Supplementary Movie 1.** **Demonstration video of the printed e-paper display.**
This movie showcases the switching behavior of the e-paper display, which is driven by the printed backplane. Various test patterns are rendered in response to the applied drive waveforms.

**Supplementary Movie 2. Demonstration of the printed OLED display.**
This movie presents a color image switching on an OLED display, operated by the printed backplane in conjunction with a white OLED layer and a color filter.
